# Fiber-Optic Temperature Sensor Using Cholesteric Liquid Crystals on the Optical Fiber Ferrules

**DOI:** 10.3390/s22155752

**Published:** 2022-08-01

**Authors:** Soyeon Ahn, Gi Hyen Lee, Jun-Yong Lee, Youngseo Kim, Min Su Kim, Srinivas Pagidi, Byeong Kwon Choi, Ji Su Kim, Jong-Hyun Kim, Min Yong Jeon

**Affiliations:** 1 Department of Physics, College of Natural Sciences, Chungnam National University, 99 Daehak-ro, Yuseong-gu, Daejeon 34134, Korea; ahnsoyen@o.cnu.ac.kr (S.A.); gtit5de@naver.com (G.H.L.); taylorseries@cnu.ac.kr (J.-Y.L.); yskim@o.cnu.ac.kr (Y.K.); kimminsu0002@gmail.com (M.S.K.); bkchoi@o.cnu.ac.kr (B.K.C.); citrus160@gmail.com (J.S.K.); jxk97@cnu.ac.kr (J.-H.K.); 2 Institute of Quantum Systems (IQS), Chungnam National University, 99 Daehak-ro, Yuseong-gu, Daejeon 34134, Korea; pagidi.srinivas@gmail.com

**Keywords:** fiber-optic sensors, temperature sensors, fiber laser, wavelength-swept laser, cholesteric liquid crystal, bandpass filter

## Abstract

Cholesteric liquid crystals (CLCs) can be applied to various physical and chemical sensors because their alignment structures are changed by external stimuli. Here, we propose a CLC device fabricated by vertically forming the helical axis of the CLC between the cross-sections of two optical fiber ferrules. An optical fiber temperature sensor was successfully implemented using the proposed optical fiber ferrule-based CLC device. A wideband wavelength-swept laser with a center wavelength of 1073 nm and scanning range of 220 nm was used as a light source to measure the variations in the reflection spectrum band according to the temperature change in the CLC cell. The wavelength variation of the reflection spectrum band according to the temperature applied to the CLC cell was reversible and changed linearly with a change in the temperature, and the long-wavelength edge variation rate according to the temperature change was −5.0 nm/°C. Additionally, as the temperature applied to the CLC cell increased, the reflection spectrum bandwidth gradually decreased; the reflection spectrum bandwidth varied at a rate of −1.89 nm/°C. The variations in the refractive indices with temperature were calculated from the band wavelengths of the reflection spectrum. The pitch at each temperature was calculated based on the refractive indices and it gradually decreased as the temperature increased.

## 1. Introduction

In the past decades, optical fibers have been extensively studied as they can be used as various sensors, such as temperature, gas, inertial navigation, displacement, and strain sensors, owing to their small sizes, flexibility, and immunity to electromagnetic and radio frequency interference [1,2,3]. Among them, fiber-based temperature sensors have been studied extensively, such as fiber Bragg grating (FBG), tapered fiber, multimode interference, hollow-core capillary waveguide, fiber Fabry–Perot interference, Mach–Zehnder interferometer, and surface plasmon resonance [4,5,6,7]. In 2018, Chunxia et al. [8] reported a tapered multi-core fiber-based temperature sensor with a sensitivity and measurable temperature range of 36.8 pm/°C and 20–100 °C, respectively. Wang et al. [9] reported a Mach–Zehnder interferometer-based temperature sensor with a sensitivity of ~8.962 nm/°C and a measurable temperature range of 33–43 °C. In 2019, Noor et al. [10] reported that the sensitivity of a temperature sensor using multimode interference generated by a single mode-multimode-single mode fiber was approximately 21 pm/°C; Han et al. [11] reported a CLC film, based on a side polished fiber environmental temperature sensor with a sensitivity of ~1.7 nm/°C and a measurable temperature range of 20–50 °C. In 2021, Liu et al. [12] reported a temperature and pressure sensor based on FBG and fiber-tip bubble, with a sensitivity of 11.1 pm/°C and a measurable temperature range of 20–100 °C.

Conversely, liquid crystals (LCs) have dielectric anisotropy and comprise soft matter that is birefringent material. Therefore, as LCs have a high sensitivity to external stimuli and have been applied to various sensors, such as temperature, electric field, and gas sensors [13,14]. A compact sensor that combines photonic crystal fiber or side-polished fiber with liquid crystal has been studied [15,16,17,18,19]. In cholesteric liquid crystals (CLCs), the directors rotate at an angle along a specific helical axis to form a periodic structure. When a circularly polarized beam with the same handedness as the CLC along the helical axis is incident, it selectively reflects only the wavelengths that satisfy the Bragg condition [20,21,22]. One period of the CLC helical structure is called the pitch. As the pitch of a CLC is easily changed by external stimuli such as heat, electric field, or magnetic field, it can be used in a variety of physical sensors [23,24,25]. Therefore, the proper combination of optical fibers and CLCs allows for the fabrication of compact and sensitive optical sensors that are easy to fabricate and operate. In 2020, Hu et al. studied a dye-doped CLC-based fiber microtip temperature sensor. The sensitivity and sensing temperature range of the sensor were approximately −9.167 nm/°C and 23–29 °C, respectively [26]. Table 1 summarizes the recent research methods for LC-based temperature sensors and their sensitivity and measurement range. Recently, we reported a temperature sensor using a ferrule-based LC cell at Photonics West 2022 [27]. In the study, anchoring was strongly observed, resulting in discontinuous pitch changes when the CLC temperature was changed; the sensitivity was approximately 1.4 nm/°C. This study reports improved results as an extension of ref. [27].

Here, we propose a CLC device that is fabricated by vertically forming the helical axis of the CLC between the cross-sections of two optical fiber ferrules. It can be used as a device for fiber-optic temperature sensors. The proposed device is easy to fabricate, inexpensive, lightweight, and compact in an all-fiber form. It is also very sensitive to temperature. The 220-nm scanning range of a broadband wavelength-swept laser (WSL) was used as a light source to measure the change in the reflection wavelength band with respect to the temperature change of the fabricated CLC cell. The characteristics of the reflection band were measured while changing the CLC cell temperature. In addition, the refractive index of the CLC was calculated using the changing characteristics of the reflection band according to the temperature change.

## 2. Fabrication of the CLC Cell

**Figure 1:**
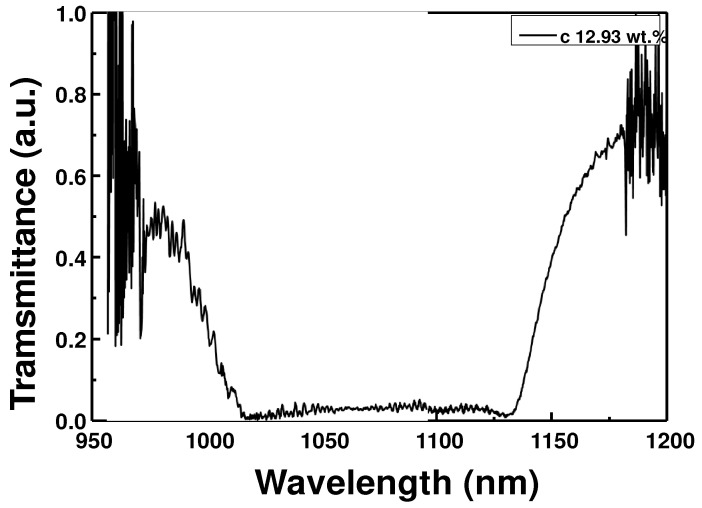
Transmission optical spectrum of CLC with a chiral dopant concentration of 12.93 wt.%.

In the experiments, a CLC cell was prepared by mixing nematic liquid crystal of E7 (Qingdao QY Liquid Crystal Co., Qingdao, China, *n_o_* = 1.5034, *n_e_* = 1.6929 @ 1100 nm) and the chiral dopant of S811 (Qingdao QY Liquid Crystal Co., HTP = 11.24 μm^−1^). The pitch of the LC can be theoretically calculated as *P* = 1/(HTP × *C*), where *P* is the pitch, and HTP and *C* are the helical twisting power and concentration of the chiral dopant, respectively [23]. Each chiral dopant has a different HTP, and the amount or tendency of pitch variation with changes as temperature differs [28,29]. The CLC cell had a horizontal orientation, in which the helical axis of the LC was perpendicular to the substrate. The prepared CLC had chiral dopant concentrations of 12.93 wt.%. After the light source of the broadband WSL was vertically incident on the fabricated optical-fiber ferrule-based CLC cell, the transmission spectra were measured to calculate the CLC pitch [24]. The transmission spectrum of the CLC cell with a chiral concentration of 12.93 wt.% is displayed in Figure 1. The short- and long-wavelength edges are 993 and 1148 nm, respectively, and the 3 dB bandwidth is approximately 155 nm. The pitch of the CLC is calculated by dividing the center wavelength of the transmission band by the average refractive index of the CLC cell [24,30]. The pitch of the CLC calculated using the transmission optical spectrum was approximately 670 nm.

**Figure 2:**
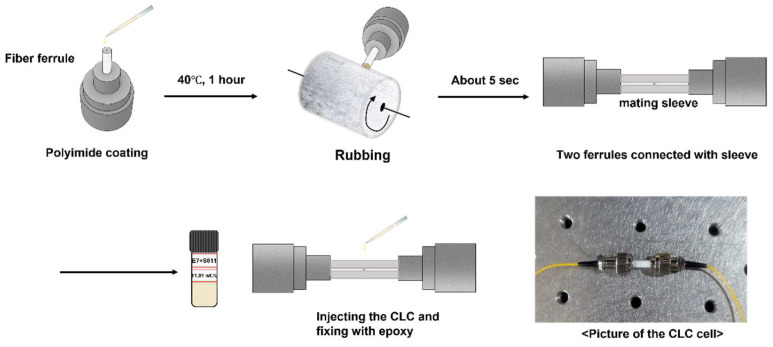
Fabrication process of the CLC cell.

The fabrication process of the CLC cell based on the fiber ferrule is portrayed in Figure 2. The fiber optic connector with ferrule used in this experiment is an FC connector. First, the cross-section of the fiber optic ferrule was cleaned with an optical fiber cleaner, and polyimide (JNC, Japan) was dropped using a pipette. Subsequently, it was placed in an oven and hardened at 40 °C for approximately 1 h. The cross-section of the optical fiber ferrule, which has been subjected to horizontal alignment, was rubbed in a specific direction such that the LC molecules were aligned in one direction. Thus, the alignment direction of the LC can be easily determined, and the reflection efficiency can be increased. The dust on the ferrule end face and mating sleeve (Thorlabs, USA, ADAF1, Ø2.5 mm) were removed using nitrogen gas and the two ferrules were fixed using the mating sleeve. In this study, a split mating sleeve was used to align the rubbing direction and a hole was drilled in the mating sleeve to allow the LC to be injected into the cell by capillary action. By rubbing the polyimide layer on the cross-section of the ferrule, the helix axis of the CLC was aligned perpendicular to the cross-section of the ferrule. After ensuring that the distance between the two ferrules was approximately several hundred micrometers, the CLC was injected using capillary action.

**Figure 3:**
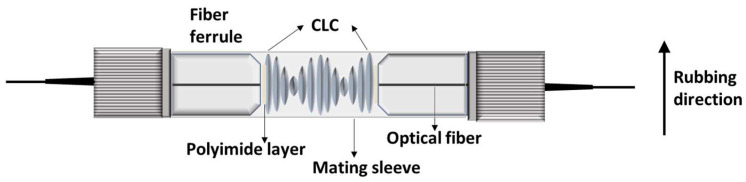
Schematic diagram of the fabricated CLC cell.

A schematic of the CLC’s helical axis arranged vertically on the cross-section of the ferrule using two optical fiber ferrules as substrates is demonstrated in Figure 3. A polyimide layer is found on each end face of the two optical fiber ferrules used as a substrate, and CLC is inserted between them. When the temperature increases, the viscosity of the LC decreases and the LC flows out through the gaps in the ferrule cell; thus, epoxy was used to close the gaps. After the epoxy was sufficiently hardened, the outer surface of the ferrule cell was coated with varnish. Because the optical fiber ferrule-based LC cell can be fabricated as an integrated optical fiber, it does not require optical alignment in free space and can be operated as a simple sensing device.

## 3. Experiments

To measure the variation in the reflection spectrum according to the applied temperature of the fabricated CLC cell, a broadband WSL was used as the light source [25]. Typically, WSLs are used as light sources in biophotonic or dynamic fiber-optic sensors [31,32,33,34,35]. However, recently, a broadband WSL with a bandwidth of at least 200 nm using two semiconductor optical amplifiers (SOAs) with different center wavelengths was realized, which proved to be useful in investigating the optical wavelength characteristics of devices in a wide wavelength band [25].

**Figure 4:**
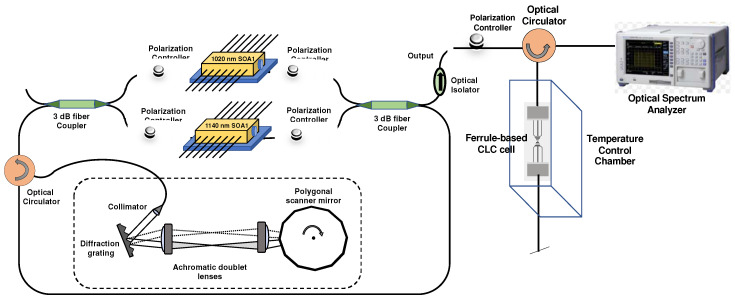
Experimental setup of the temperature sensor using wideband WSL.

**Figure 5:**
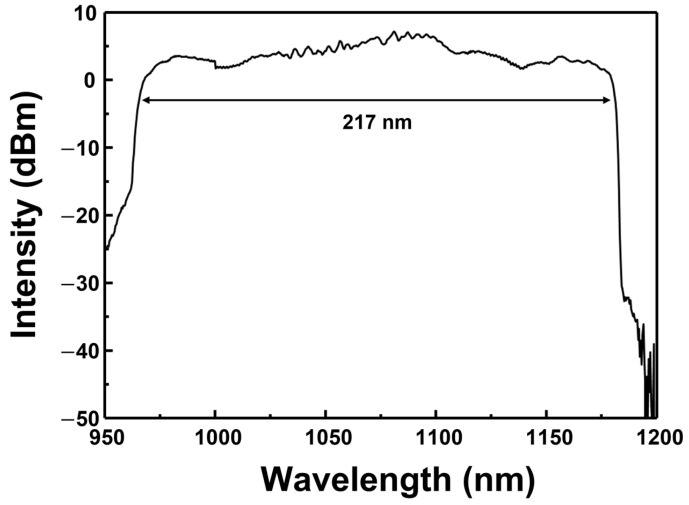
Optical spectra of the WSL.

The experimental setup of the fiber-optic temperature sensor using a wideband WSL is demonstrated in Figure 4. The wideband WSL consisted of two SOAs (Innolume Inc., Germany), two 3 dB-fiber couplers, an optical circulator, four polarization controllers (PCs), and a polygonal mirror scanning wavelength filter (PMSWF). The optical fibers used in the experiment are SM-980 and HI-1060 single-mode fibers. The center wavelengths of the amplified spontaneous emission of the two SOAs were 1020 and 1040 nm, and the 10-dB bandwidths were 114 and 57 nm, respectively. The dotted box in Figure 4 portrays the PMSWF, which comprised a fiber-optic collimator, brazed diffraction grating, two achromatic doublet lenses, and a polygonal scanner mirror (Cambridge Technology Inc., USA). A WSL with a central wavelength of 1073 nm and a 10-dB bandwidth of approximately 217 nm (from 965 to 1182 nm) was used, as demonstrated in Figure 5. The average intensity of the WSL was approximately 13.8 mW at a scanning frequency of 1.8 kHz. The output of the WSL passed through an optical isolator and a PC, and then entered the CLC device in the temperature control chamber (LCH-11, Jeiotech, Korea, fluctuation: ±0.3 °C, temperature range: −20–100 °C) through an optical circulator. The connector of the CLC cell of the sensor unit is the FC/UPC type and the optical fiber is the SM-980 single-mode fiber. The reflection spectrum of the reflected beam from the CLC device through the optical circulator was measured using an optical spectrum analyzer (OSA; Yokogawa, Japan, Ando AQ6317B). As a PC reflects circularly polarized light in the same direction as the twisted direction of the CLC, it was used to increase the reflectance. The CLC device was placed in a temperature-controlled chamber and the variation in the reflection spectrum owing to temperature change was measured.

**Figure 6:**
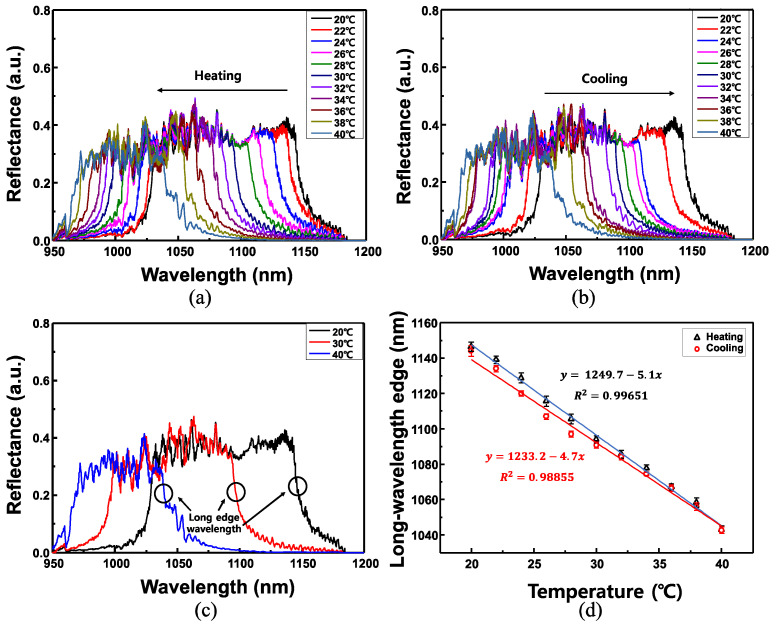
(**a**) Change in reflection spectra when the temperature of the CLC cell is increased from 20 to 40 °C, (**b**) change in reflection spectra when the temperature of the CLC cell is decreased from 40 to 20 °C, (**c**) reflection spectra for several temperatures, and (**d**) a graph of the variation in wavelength when the CLC cell was heated and cooled.

The reflected spectra from the CLC device were measured by changing the temperature from 20 to 40 °C with intervals of 2 °C. Five heating and cooling cycles were employed to validate the repeatability. The CLC used in the experiment had a chiral dopant concentration of 12.93 wt.%, a pitch of approximately 670 nm, and a distance between the ferrule cross-sections of the CLC device of several hundreds of micrometers. Each temperature was measured at a stabilization time of 10 min. In the CLC used in the experiments, the temperature at which the CLC phase changed to the isotropic phase was approximately 53 °C and the temperature variation can be measured until the CLC phase is maintained. In this study, however, when the temperature of the CLC increased above 40 °C, it could not be measured anymore because it deviated from the measurable spectrum range (965~1182 nm) of the light source. The variations in the reflection spectra according to temperature variations are portrayed in Figure 6a,b. These changes occurred in the reflection spectra when the temperature of the CLC cell was increased from 20 to 40 °C and when the temperature was decreased from 40 to 20 °C. As portrayed in Figure 6a, when the CLC cell is heated, the reflection band spectrum moves toward the shorter wavelength. Conversely, as demonstrated in Figure 6b, the reflection band spectrum moves to a longer wavelength when the CLC cell is cooled. The reflection spectra at several temperatures are portrayed in Figure 6c. The long-wavelength edges and bandwidths at 20, 30, and 40 °C are 1150.0, 1098.8, and 1045.6 nm, and ~122, ~102, and ~79 nm, respectively. In Figure 6c, the long-wavelength edges at each temperature are indicated by circles. A graph of the variation in the long-wavelength edge of the reflection spectrum band for the heating and cooling applied to the CLC cell is portrayed in Figure 6d. The liquid crystal undergoes a variation in refractive index and pitch according to the temperature change and a phenomenon in which the reflection band shifts occur. The refractive index of the CLC changes more in the long-wavelength edge-related $n_{e}\;$ than the short-wavelength edge-related $n_{o}$ according to the temperature change; thus, the long-wavelength edge with a larger wavelength variation at the same temperature change was selected. Here, the measured long-wavelength edge was approximately 60% of the spectral width of the reflection band. This was set to 60% to minimize the error owing to the jitter of the reflection band peak. As observed, the change in wavelength is reversible and changes linearly with temperature. The rate of change of the long-wavelength edge with temperature was −5.1 nm/°C and −4.7 nm/°C for heating and cooling, respectively. In addition, thermal hysteresis was observed, which may result from the degradation of CLC at high temperatures [36]. The small hysteresis may be because the temperature range of the sensor was realized at a lower temperature compared with the nematic-isotropic transition temperature (T_NI) of CLC (~53 °C). The measured range of the implemented sensor is 20–53 °C; however, in this experiment, it was limited to 20–40 °C, owing to the spectrum limitation of the measured light source. The sensitivity of the proposed temperature sensor was approximately −5.0 nm/°C. Owing to the temperature chamber used in the experiment, the detection limit was ±0.3 °C.

**Figure 7:**
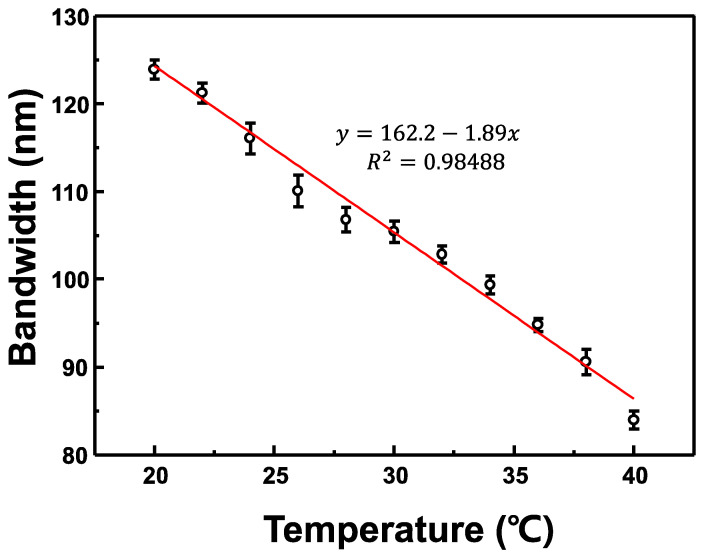
Graphs of the reflection spectrum bandwidth of the CLC cell with changes in temperature.

The graphs of the variation in the reflection spectrum bandwidth of the CLC cell with changes in temperature are portrayed in Figure 7. The error bar was calculated from the standard error and the measurement was repeated five times. When the temperature of the CLC cell was 20 °C, the spectral bandwidth was ~124 nm, whereas when the temperature of the CLC cell was increased to 40 °C, the spectral bandwidth decreased to ~84 nm. A linear relationship was observed as the temperature applied to the CLC cell increased and the reflection spectrum bandwidth gradually narrowed. This is because the birefringence of the CLC decreased as the temperature of the CLC cell increased. The rate of variation of the bandwidth of the reflection spectrum with temperature change was ~−1.89 nm/°C as portrayed in Figure 7.

The refractive indices of the CLC cell can be calculated using the variations in wavelength with temperature. The variation in the refractive indices of the CLC with temperature is expressed as follows [37,38]:

(1) $$n_{e}\left(T\right)≅A-BT+\frac{2{\left(\Delta n\right)}_{o}}{3}{\left(1-\frac{T}{T_{c}}\right)}^{\beta }$$

(2) $$n_{o}\left(T\right)≅A-BT-\frac{{\left(\Delta n\right)}_{o}}{3}{\left(1-\frac{T}{T_{c}}\right)}^{\beta },$$

where $n_{o}\;\mathrm{and}\;n_{e}$ are the ordinary and extraordinary refractive indices of the LC, respectively; *T* is the absolute temperature; *T_c_* is the transition temperature from nematic to isotropic LC; *β* is the material constant; *A* and *B* are constants; ${\left(\Delta n\right)}_{o}$ is the birefringence of the LC at *T =* 0 K. Further, the average refractive index $\langle n\rangle$ of the LC is expressed as follows: [39]

(3) $$\langle n\rangle =\frac{n_{e}+2n_{o}}{3}.$$

The relationships between wavelength and refractive indices are expressed as follows:

(4) $$\lambda_{s}=n_{o}P\;,\;\lambda_{l}=n_{e}P,$$

where $\lambda_{s}$ and $\lambda_{l}$ are the short- and long-wavelength edges of the reflection spectrum band, respectively. The wavelengths of $\lambda_{s}\;$ and $\lambda_{l}$ are obtained from the reflection spectrum band and the four unknowns, *A*, *B*, ${\left(\Delta n\right)}_{o}$, and *β* are obtained from the nonlinear fitting using the following ratio of $n_{o}$ and $n_{e}$:

(5) $$\frac{\lambda_{s}}{\lambda_{l}}=\frac{n_{o}}{n_{e}}=\frac{A-BT-\frac{{\left(\Delta n\right)}_{o}}{3}{\left(1-\frac{T}{T_{c}}\right)}^{\beta }}{A-BT+\frac{2{\left(\Delta n\right)}_{o}}{3}{\left(1-\frac{T}{T_{c}}\right)}^{\beta }}.$$

**Figure 8:**
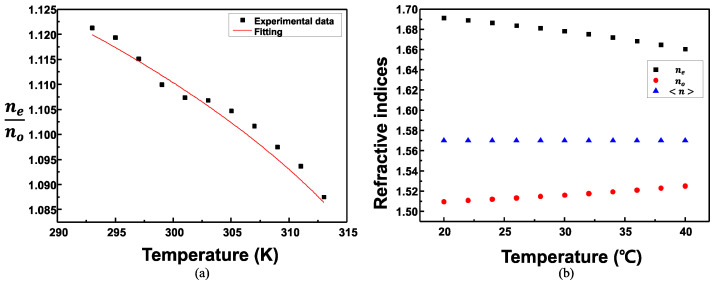
(**a**) Graph of nonlinear fitting using the experimental data of the ratio of $n_{o}$ and $n_{e},$ and (**b**) the refractive indices of $n_{o}$, $n_{e}$, and $\langle n\rangle$ according to the temperature obtained by applying the four constant values.

A graph of the nonlinear regression fitting using the experimental data for the ratio of $n_{o}$ and $n_{e}$ is demonstrated in Figure 8a. The constant values of *A*, *B*, ${\left(\Delta n\right)}_{o}$, and *β* obtained from the nonlinear regression fitting graph are listed in Table 2. The constant values of *A, B,* ${\left(\Delta n\right)}_{o}$, and *β* were obtained as 1.5072, 6.4173 × 10^−8^, 0.3747, and 0.3159, respectively. The refractive indices of $n_{o}$, $n_{e}$, and $\langle n\rangle$ according to the temperature obtained by applying the four constant values are portrayed in Figure 8b. As the temperature of the CLC cell increases, $n_{e}$ decreases gradually; however, $n_{o}$ instead increased gradually. Consequently, the average refractive index of $\langle n\rangle$ was 1.57, which was almost the same; however, it slightly decreased as the temperature increased [38].

From the refractive indices of $n_{o}$ and $n_{e}$, the varying pitch according to the temperature applied to the CLC cell may be calculated. The relationship between the central wavelength $\lambda_{c}$ of the reflection spectrum band and pitch *P* of the CLC cell is expressed as follows:

(6) $$P=\frac{2}{n_{o}+n_{e}}\lambda_{c}$$

**Figure 9:**
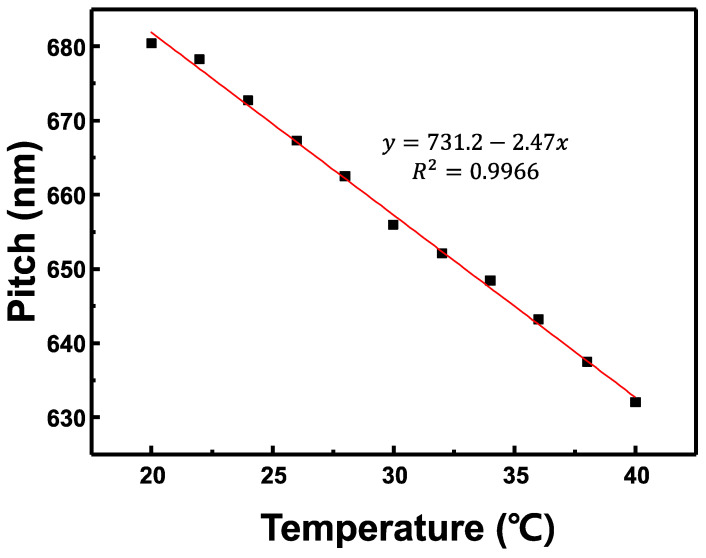
Variation of the pitch of the CLC cell according to the temperature calculated from the refractive indices in Figure 8.

The variation of the pitch of the CLC cell with the temperature calculated from the refractive indices in Figure 8 is portrayed in Figure 9. The pitch of the CLC cell measured at 25 °C was ~670 nm and the calculated pitch at 24 °C using the refractive index was approximately 672 nm. The measured and calculated values were almost equal. The rate of change of the pitch with respect to the temperature of the CLC cell was −2.42 nm/°C.

## 4. Conclusions

We proposed a CLC device based on a fiber ferrule and successfully demonstrated a CLC-based optical fiber temperature sensor. A 220-nm broadband wavelength-swept laser was used as the light source to measure the variations in the reflection spectrum band with the temperature applied to the CLC in a wide wavelength band of approximately 1073 nm. As the temperature of the CLC cell increased, the long-wavelength edge of the reflection band shifted to a shorter wavelength. The relationship between the temperature and center wavelength variation of the reflection band was almost linear. The long-wavelength edge variation rate with the temperature change of the CLC cell was −5.0 nm/°C. As the temperature applied to the CLC cell increased, the reflection spectrum bandwidth gradually decreased; the reflection spectrum bandwidth variation rate was −1.89 nm/°C. In addition, the variations in refractive indices with temperature were calculated from the wavelength of the reflection spectrum band. When the pitch of the CLC cell at each temperature was calculated based on the refractive indices, the pitch exhibited a gradually decreasing tendency as the temperature increased. In the future, a simple fabrication process is expected for the proposed CLC cells, and the cells are expected to be utilized in various fiber-optic sensors.

## Figures and Tables

**Table 1:** Comparison of recent research results.

Authors	Year	Method	Sensitivity	Detection Range
Chunxia et al. [8]	2018	tapered multi-core fiber	36.8 pm/°C	20~1000 °C
Wang et al. [9]	2018	Mach–Zehnder interferometer	8.962 nm/°C	33~43 °C
Noor et al. [10]	2019	multi-mode interference	21 pm/°C	-
Han et al. [11]	2019	CLC film based on a side polished fiber	1.7 nm/°C	20~50 °C
Hu et al. [26]	2020	dye-doped CLC-based on a fiber micro tip	−9.167 nm/°C	23~29 °C
Liu et al. [12]	2021	FBG and fiber-tip bubble	11.1 pm/°C	20~100 °C
Our work	2022	Optical fiber ferrule-based CLC cell	−5.0 nm/°C	20~40 °C

**Table 2:** Constant values of *A*, *B*, ${\left(\Delta n\right)}_{o}$ , and *β*.

*A*	*B*	${\left(\Delta n\right)}_{o}$	β
1.5072	6.4173 × 10^−8^	0.3747	0.3159

## Data Availability

Data available on request from the authors.
